# Erythrocyte hemighosts in a patient with tumor lysis syndrome: One train may hide another

**DOI:** 10.1002/ccr3.3011

**Published:** 2020-07-07

**Authors:** Pierre Cabantous, Sylvie Daliphard, Victor Bobée

**Affiliations:** ^1^ Department of Biological Hematology Rouen University Hospital Rouen France

**Keywords:** G6PD deficiency, hematology, hemolytic anemia, methemoglobin, venetoclax

## Abstract

Rasburicase was introduced to treat hyperuricemia secondary to tumor lysis syndrome. Because of severe hemolytic anemia, a blood smear was requested and showed hemighosts, revealing G6PD deficiency. Erythrocyte morphology is a key tool in laboratory hematology.

## Graphical abstract

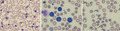

A 50‐year‐old man was diagnosed with chronic lymphoid leukemia with trisomy 12 and deletion 17p. A rapid increase of the lymphocyte count led to the decision to treat, and venetoclax was introduced. The patient rapidly developed tumor lysis syndrome with hypocalcemia, hyperphosphoremia, and hyperuricemia (493 µmol/L) and was treated by rasburicase and hyperhydration. Oxygen desaturation at 70% was found together with methemoglobinemia of 9.4%, thrombocytopenia of 112 × 10^9^/L and severe hemolytic anemia (hemoglobin 5.6 g/dL, reticulocytes 78 × 10^9^/L, low haptoglobin, LDH 2905 U/L). A blood smear was requested by laboratory hematologist and revealed red cells with irregular hemoglobin repartition and damaged membrane, also known as hemighosts (Figure 1, Panel A‐C), a hallmark of severe oxidative injury frequently found in patients with glucose‐6‐phosphate dehydrogenase (G6PD) deficiency. Diagnosis was confirmed by enzymatic dosage, and treatment was switched to allopurinol. Detailed patient interview revealed Caribbean‐born ascendants, and molecular analysis showed G6PD A‐ mutant.

**Figure 1:**
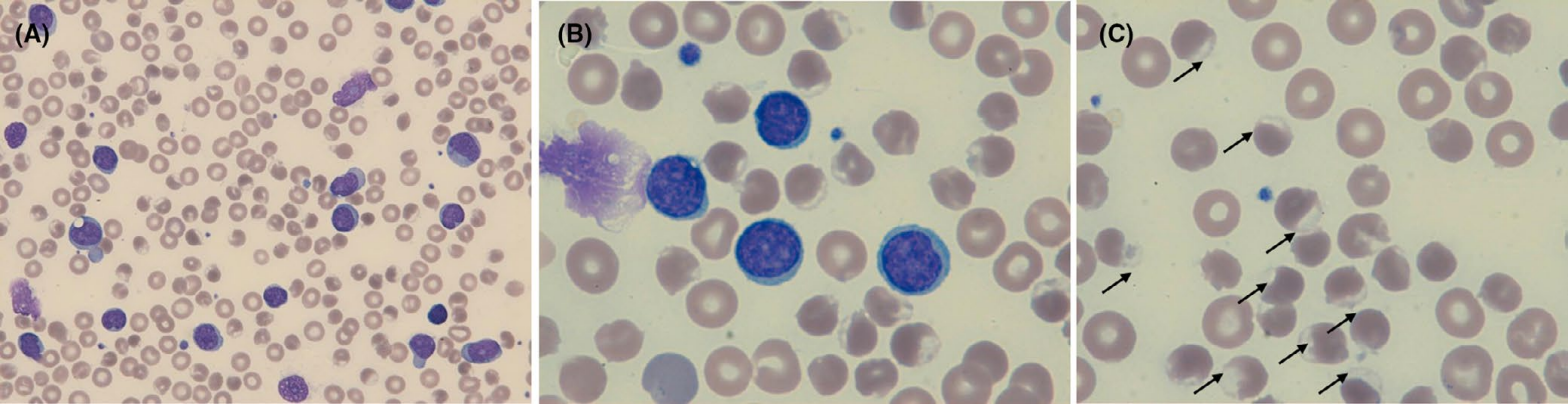
Blood smear of the patient. The blood smear shows numerous lymphocytes and lysed cells in accordance with the CLL diagnosis, as well as red blood cells with damaged membrane and a clear peripheral zone called hemighosts (arrows in panel C). May‐Grünwald‐Giemsa staining. A, total magnification ×500. B‐C, total magnification ×1000

Erythrocyte morphology was essential to evoke G6PD deficiency in emergency. Rasburicase is an oxidative drug contraindicated in G6PD deficiency that induced intravascular hyperhemolysis and promoted methemoglobin formation^1^ through oxidation of heme iron to the ferric state. G6PD enzymatic dosage should be considered before rasburicase introduction.^2^

## CONFLICT OF INTEREST

None.

## AUTHOR CONTRIBUTIONS

PC: performed research and analyzed data. SD: performed research and analyzed date. VB: coordinated the study, wrote the paper, and supervised analysis.
